# Supplementary material for: From blueprint to biobank: Leveraging expert recommendations for implementing change (ERIC) to pediatric cancer biobanking in Pakistan
Source: PLoS One. 2025 May 16;20(5):e0321316. doi: 10.1371/journal.pone.0321316 (PMC12083815; doi:10.1371/journal.pone.0321316)
Supplement: S1 Table — (DOCX) [file pone.0321316.s001.docx]

| **Available items** | **Procured items** |
| --- | --- |
| Biosafety cabinet | Cool cell LX cell freezing container |
| LUNA-FL™ dual fluorescence cell counter | Cryo sleeves |
| Pipettes (1000 µL, 200 µL, 10 µL) | Cryo canes |
| Pipette aid machine | 50 Litre liquid nitrogen tank-Qty: 02 |
| Vortex mixer | 10 Litre liquid nitrogen tank-Qty: 03 |
| 50 mL centrifuge | Cryo gloves resistant LN2 for liquid nitrogen |
| 2 mL micro centrifuge | Liquid nitrogen measuring stick |
| -80 ◦C freezer | Liquid nitrogen hand pump |
| 2-8 ◦C refrigerator | Liquid nitrogen digital level monitor |
| Ice maker machine | Wireless temperature data loggers |
|  | Liquid nitrogen enclave:  ● Access control system  ● Air balancing monitoring system  ● Exhaust fan  ● Electrical fixtures  ● HVAC system  ● Civil works |

**S1 Table:** List of items available and procured, respectively, for pediatric acute leukemia biobanking with the clinical laboratory of Indus Hospital & Health Network.
