# Supplementary material for: From blueprint to biobank: Leveraging expert recommendations for implementing change (ERIC) to pediatric cancer biobanking in Pakistan
Source: PLoS One. 2025 May 16;20(5):e0321316. doi: 10.1371/journal.pone.0321316 (PMC12083815; doi:10.1371/journal.pone.0321316)
Supplement: S2 Table — (DOCX) [file pone.0321316.s002.docx]

| **Markers** | **PBMC thawed specimens** | **Diagnostic specimens** |  |  |
| --- | --- | --- | --- | --- |
|  | **Strength (Mean Fluorescent Intensity)** | **Strength (Mean Fluorescent Intensity)** | **Diagnostic-Thawed Strength Diff** | |
| **FSC-A** | 9328 | 72724 | -63396 | |
| **SSC-A** | 32433 | 16145 | 16288 | |
| **CD 45** | 2042 | 3670 | -1628 | |
| **CD 41** | 3457 | 1193 | 2264 | |
| **CD 61** | 4199 | 5212 | -1013 | |
| **CD 42b** | 1309 | 1247 | 62 | |
| **HLADR** | 514 | 517 | -3 | |
| **CD 36** | 4069 | 2823 | 1246 | |
| **IC CD 3** | 343 | 409 | -66 | |
| **CD16** | 216 | 117 | 99 | |
| **CD33** | 438 | 567 | -129 | |
| **CD34** | 405 | 401 | 4 | |
| **CD19** | 216 | 135 | 81 | |
| **CD117** | 122 | 186 | -64 | |
| **CD14** | 119 | 158 | -39 | |
| **CD13** | 186 | 780 | -594 | |
| **CD4** | 1979 | 846 | 1133 | |
| **Glycophorin A** | 286 | 350 | -64 | |
| **HLA-DR** | 614 | 157 | 457 | |
| **TdT** | 410 | 212 | 198 | |
| **MPO** | 359 | 394 | -35 | |
| **IC CD 79a** | 294 | 86 | 208 | |
| **FSC-A** | 83674 | 40265 | 43409 | |
| **SSC-A** | 21215 | 12400 | 8815 | |
| **IC CD 3** | 3646 | 2705 | 941 | |
| **IC TdT** | 763 | 937 | -174 | |
| **CD79a** | 181 | 156 | 25 | |
| **IC CD3** | 3646 | 2705 | 941 | |
| **CD13** | 476 | 128 | 348 | |
| **SF CD 3** | 5959 | 1310 | 4649 | |
| **CD 8** | 677 | 1340 | -663 | |
| **CD 4** | 337 | 451 | -114 | |
| **CD 2** | 8534 | 1445 | 7089 | |
| **CD 45** | 2202 | 2600 | -398 | |
| **CD 7** | 18113 | 4386 | 13727 | |
| **CD 99** | 1502 | 681 | 821 | |
| **IC MPO** | 275 | 516 | -241 | |
| **CD33** | 476 | 128 | 348 | |
| **CD123** | 1526 | 1588 | -62 | |
| **CD73** | 400 | 414 | -14 | |
| **CD5** | 961 | 1404 | -443 | |
| **CD4** | 337 | 451 | -114 | |
|  |  | **Mean Difference** | **807.12** | |
|  |  | **St.Dev** | **12586.55** | |
|  |  | **Sig. Level** | **0.05** | |
|  |  | **Sample #** | **42.00** | |
|  |  | **95% CI** | **3806.54** | |
|  |  | **Upper CI value** | **4613.66** | |
|  |  | **Lower CI value** | **-2999.42** | |

**S2 Table**: Post-thaw PBMC cell viability and surface marker expression were tested through flow cytometric analysis. These were compared to those obtained with fresh whole blood specimens at the time of diagnostic flow cytometry. 95% CI of the mean difference of the marker expression strength included zero, signifying no statistically significant difference between positive marker expression in diagnostic and post-thaw specimens.
