# Supplementary figures and images for: From blueprint to biobank: Leveraging expert recommendations for implementing change (ERIC) to pediatric cancer biobanking in Pakistan
Source: PLoS One. 2025 May 16;20(5):e0321316. doi: 10.1371/journal.pone.0321316 (PMC12083815; doi:10.1371/journal.pone.0321316)

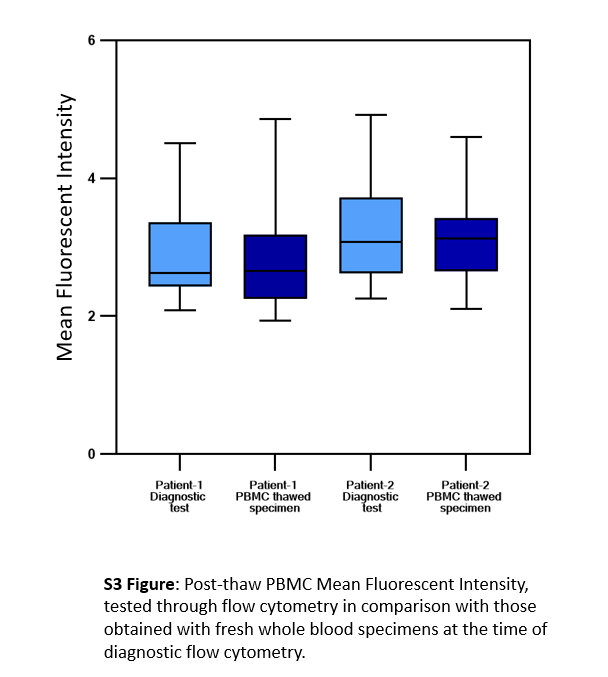

Supplement: S1 Fig — (TIF) [file pone.0321316.s003.tif]
