# Supplementary material for: From blueprint to biobank: Leveraging expert recommendations for implementing change (ERIC) to pediatric cancer biobanking in Pakistan
Source: PLoS One. 2025 May 16;20(5):e0321316. doi: 10.1371/journal.pone.0321316 (PMC12083815; doi:10.1371/journal.pone.0321316)
Supplement: Attachment — Submitted filename: Review From Blueprint to Biobank Biobanking in Pakistan.docx [file pone.0321316.s005.docx]

**Review: From Blueprint to Biobank: Leveraging Expert Recommendations for Implementing Change (ERIC) to Pediatric Cancer Biobanking in Pakistan**

Thank you very much for giving me the opportunity to review this article.

**Summary:**

In this study, authors aim to provide a description of the biobank implementation process in Pakistan utilizing implementation science framework. In a country with restricted resources and limited prior experiences, authors discuss the implementation of Pakistan’s first acute leukemia biobank using ERIC and EPIS frameworks as structured approach. For this process, specimens were collected while the workgroups in implementation planning included clinicians, EMR, IT, management, senior leadership, IRB, and external support from UNC and St. Jude Children’s Cancer Hospital. Authors provide the details of process which was carried out utilizing strategies such as ERIC (Expert Recommendations for Implementing Change). Furthermore, The EPIS framework (exploration, preparation, implementation, sustainment) was utilized to map and organize the selected intervention strategies.

**Comment:**

Overall, it is a novel study, relevant to the field. This study describes various challenges related to effective biospecimen management. By improving implementation strategies as described by authors, pediatric cancer research can benefit from more efficient and effective biospecimen management, ultimately contributing to better research outcomes and patient care.

I have a few questions. I believe briefly addressing these points will enhance the quality of this manuscript.

Authors may want to comment on the following.

- Do authors envision data sharing and collaboration at international level? If so, authors need to comment on how this model may deal with potential problems such as, data protection laws, and logistical issues.
- Authors need to be more explicit about ethical and legal considerations. Authors have mentioned some related issues briefly. However, they have not discussed the challenges of building trust and engaging with the community and stakeholders, which is essential for the success of a biobank. In fact in a country of limited resources, the ethical issues could be enormous. There is a risk of exploiting vulnerable populations, such as those in desperate financial situations, by offering monetary compensation for plasma donations. This can lead to individuals donating more frequently than is safe, potentially compromising their health, maintaining high standards of safety and quality in plasma collection and storage can be challenging in resource-limited settings. This includes ensuring proper sterilization, handling, and storage to prevent contamination and degradation of plasma, Inadequate regulatory frameworks and enforcement can lead to unethical practices and compromised safety standards. Ensuring that plasma collection centers adhere to strict guidelines and are regularly inspected is essential. Authors may briefly address these points as well.

.

- Financial constraints, funding and effective education of patient/participant in this area are going to be a challenge to implement this strategy nationwide, especially in rural areas. In Pakistan where a majority of population resides in rural areas, practical implication of this strategy may be a challenge.
